# Impact of Flaxseed Gums on the Colloidal Changes and In Vitro Digestibility of Milk Proteins

**DOI:** 10.3390/foods11244096

**Published:** 2022-12-18

**Authors:** Thierry Hellebois, Jennyfer Fortuin, Claire Gaiani, Christos Soukoulis

**Affiliations:** 1 Environmental Research and Innovation (ERIN) Department, Luxembourg Institute of Science and Technology (LIST), L-4362 Esch-sur-Alzette, Luxembourg; 2 Laboratoire d’Ingénierie des Biomolécules (LIBio), Université de Lorraine, F-54000 Nancy, France; 3 Food Quality and Design Group, Wageningen University and Research, 6708 NL Wageningen, The Netherlands

**Keywords:** *Linum usitatissimum* L., mucilage, in vitro digestion, whey protein, sodium caseinate, protein–polysaccharide interactions

## Abstract

Flaxseed (*Linum usitatissimum* L.) mucilage is one of the most studied plant seed gums in terms of its techno-functional and health-promoting properties. Nonetheless, the interplay of flaxseed gum (FG) with other food biopolymers, such as milk proteins, under in vitro digestion conditions remains underexplored. The aim of the present work was to investigate the colloidal interplay between flaxseed gum (golden or brown) and milk proteins (sodium caseinate or whey protein isolate) under simulated in vitro digestion conditions and its relationship with the attained in vitro protein digestibility. The presence of flaxseed gum in the milk protein food models and in the oral food boluses obtained was associated with the occurrence of segregative microphase separation. Flaxseed gum exhibited a prominent role in controlling the acid-mediated protein aggregation phenomena, particularly in the sodium caseinate gastric chymes. The addition of FG in the food models was associated with a higher amount of intact total caseins and β-lactoglobulin at the end of the gastric processing step. Monitoring of the intestinal processing step revealed a very advanced cleavage of the whey proteins (>98%) and caseins (>90%). The degree of the milk protein hydrolysis achieved at the end of the intestinal processing was significantly higher in the systems containing flaxseed gum (i.e., 59–62%) than their gum-free protein counterparts (i.e., 46–47%). It was postulated that the electrostatic milk protein complexation capacity and, to a lesser extent, the thickening effect of flaxseed gum influenced the in vitro digestibility of the milk proteins.

## 1. Introduction

Flax (*Linum usitatissimum* L.) is one of the most important industrial crops cultivated for its fibres and seeds. Although flax fibres are prevalently exploited in the manufacturing and composite biomaterials domain [1], flaxseed is considered a rapidly emerging food biomass in the production of sustainable dietary relevant ingredients such as soluble dietary fibres (mucilage), proteins, and lipids [2]. Its mucilaginous matter, located in the outermost seed layer, accounts for 3–9% of the weight of the whole seed. From an osidic composition viewpoint, flaxseed gum (FG) comprised three major polysaccharidic fractions: a low molecular weight rhamnogalacturonan-I (RG-I) rich (M_w_ ~300–700 kDa), a high molecular weight arabinoxylan (AX) rich (M_w_ ~2000–4000 kDa), and a medium molecular weight (M_w_ ~700–1500 kDa composite heteropolymeric (RG-I and AX) fraction [3]. Other than the phenotypic and genotypic characteristics of flaxseed, the aqueous extraction conditions, i.e., temperature, pH, ionic strength, seed-to-water ratio, the implementation of extraction-aiding physical processing (e.g., ultrasound or microwave), and the gum purification and dehydration methods determine the structure conformation and consequently, the inherent techno-functional properties (e.g., thickening, gelling, stabilising) of flaxseed gums [4].

Milk proteins i.e., caseins (α_s_-, β- and κ-) and whey proteins (primarily β-lactoglobulin, α-lactalbumin, lactoferrin, immunoglobulins, and proteose peptones), possess a substantial techno-functional and dietary role in food product design [5]. From a nutritional viewpoint, milk proteins are widely recognised as natural sources of essential amino acids and bioactive peptides [6]. Due to their inherent structure conformational dissimilarity, milk proteins exert a diversified colloidal response to intragastric conditions (i.e., acid and pepsin-induced coagulation), which in turn can impact their resistance to the peptic cleavage that takes place during gastrointestinal transit. Due to their loose and more flexible structure, caseins undergo extensive intragastric coagulation compared to the well-defined globular structure conformation of whey proteins [6,7]. On the one hand, α_s_-, β- and κ-caseins are less resistant to peptic cleavage than α-lactalbumin and β-lactoglobulin as a result of the selectivity of pepsin for the looser structure of the former. On the other hand, it is well documented that the ingestion of caseins favours the formation of large aggregates in the stomach, impeding gastric emptying, and thus, caseins exhibit a more sustained postprandial aminoacidemic response than whey proteins [8]. Other parameters associated with food processing, such as homogenisation, and enzymatic or thermal pretreatment, also have been reported to influence the digestibility of milk proteins [6,9,10,11].

Polysaccharides play a substantial role in the food industry, serving as natural sources of dietary fibre and as structuring, texturising, thickening, and stabilising agents [12]. It is well documented that mixing milk proteins with polysaccharides may result in associative (complex coacervation) or non-associative segregative phase separation phenomena [13]. The colloidal interplay between milk proteins and polysaccharides impacts the textural and structural aspects of the food matrix and modulates its colloidal configuration throughout oro-gastrointestinal transit [14]. In this context, the presence of polysaccharides in proteinaceous-rich food matrices may sustain their disintegration rate and suppress the susceptibility of milk proteins to peptic cleavage [15,16,17,18,19,20]. Borreani and co-workers [15] demonstrated that regardless of the protein source (i.e., skim milk powder, whey protein isolate, or sodium caseinate), anionic polysaccharides (sodium alginate) exerted a better ability to hinder the intragastric pepsinolytic activity than the non-ionic exemplars (konjac gum). Studying binary blends of sodium alginate with microparticulated whey protein, Koutina and co-workers [17] reported that the degree of the peptic cleavage of whey proteins was adversely associated with the molecular weight of sodium alginate, which was ascribed to the formation of protein-polysaccharide coacervates. Implementing a semi-dynamic in vitro digestion model, Markussen and co-workers [20] demonstrated that the occurrence of segregative phase separation and complex coacervation phenomena are the major drivers of the intragastric-induced peptic cleavage of milk protein concentrate (MPC). In a recent study, Hellebois and co-workers [21] demonstrated that alfalfa seed galactomannan modulated the acid-induced aggregation of milk proteins, leading to higher rates of free amino group release in the intestinal digesta.

Notwithstanding the broad techno-functionality of flaxseed gums, their interplay with proteins under in vitro digestion-simulating conditions remains unexplored. The present study aimed to understand the impact of two different types of flaxseed gums (brown and golden) on the in vitro digestibility of two milk protein (whey protein isolate and sodium caseinate) liquid food models. From a mechanistic standpoint, it is hypothesised that flaxseed gum, due to its anionic character, can modulate the peptic cleavage of milk proteins through its ability to control the acid-induced protein aggregation phenomena and hinder the proteases diffusivity sterically at the solid–liquid interface boundaries.

## 2. Materials and Methods

### 2.1. Materials

Whey protein isolate (WPI) PRODIET 90S, with a protein content of 87.7% wt., was kindly donated by Ingredia (Arras, France). Sodium caseinate (NaCN, protein purity of 89.4% wt.) was obtained from Sigma–Aldrich (Leuven, Belgium). The exact protein content of the milk proteins was determined using the Dumas method (Elementar Vario Cube, Langensenbold, Germany). The gum extraction and isolation from golden and brown flaxseed (GF and BF, respectively) was conducted at pH = 8 following the method explicitly described by Hellebois and co-workers [3]. All the other chemicals used were purchased from Sigma–Aldrich and were of analytical grade.

### 2.2. Preparation of the Milk Protein Food Models

The appropriate amount of protein powder was dissolved in ultrapure water (18.2 mΩ, Merck Millipore, Burlington, MA, United States) to achieve a protein concentration of 10% wt. and was stirred until complete dissolution. Following the complete dissolution of the protein, the solution was heat treated at 80 °C for 20 min in a shaking water bath (SW22, Julabo, Seelbach, Germany). The solution obtained was then homogenised three times using a high-pressure homogeniser (Panda plus 2000, GEA, Düsseldorf, Germany) at 500 bar before being centrifuged at 18,500× *g* for 10 min to remove the non-solubilised particles. The homogeneity of the protein suspension was confirmed using laser light scattering particle size analysis (Mastersizer 3000, Malvern Instruments, Worcestershire, United Kingdom). To prevent bacterial growth, 0.02% wt. sodium azide (NaN_3_) was added. BF or GF was then added to the solution to achieve 0.1, 0.5, and 1.0% wt. and the solution was solubilised overnight. Both WPI and NaCN solutions were prepared using this method.

### 2.3. In vitro Gastrointestinal Processing of the Milk Protein Food Models

The in vitro digestion experiment was based on the INFOGEST protocol [22], as previously employed in protein-polysaccharide biopolymer systems. Briefly, ten millilitres of the initial matrices (WPI/BF or GF; NaCN/BF or GF) were diluted 1:1 with simulated saliva fluids containing 75 U mL^−1^ of α-amylase and incubated for 3 min in a shaking water bath (SW22, Julabo, Seelbach, Germany) at 37 ± 0.1 °C, 100 min^−1^. The oral bolus was then diluted 1:1 with simulated gastric fluids containing 2000 U mL^−1^ of pepsin, adjusted with HCl 1 M at pH = 2.5 and incubated for 2 h at 37 °C ± 0.1 °C, *f* = 100 min^−1^. The intestine phase was initiated by diluting 1:1 the gastric chymes with simulated intestine fluids containing the bile salts and 100 U mL^−1^ of pancreatin and was then adjusted to pH = 7 with NaOH 1 M. Samples were collected in the initial food matrix at the end of the oral phase and at t = 0, 5, 10, 20, 30, 60, 90, and 120 min in the gastric and intestinal phases. The proteases activity was ceased by the addition of a protease inhibitor mix (Cytiva, Marlborough, MA, USA), and the samples were either kept in ice for direct analysis or quench frozen in liquid nitrogen and kept at −80 °C until use.

### 2.4. Steady State Flow Rheological Measurements

The initial food matrices and the oral, gastric, and intestinal phases were rheologically assessed. The flow behaviour of the systems was measured at a shear rate from 0.1 to 1000 s^−1^ at 37 °C using a double gap (DG 26.7) geometry of 27.1 mm diameter mounted on an oscillatory rheometer (MCR 302, Anton Paar, Graz, Austria). The apparent viscosity of the food matrices and digesta was measured at 50 s^−1^. The contribution of the sedimentable aggregates formed during the gastric phase on the rheological properties was assessed by measuring the soluble fraction after a centrifugation step at 4500× *g* for 5 min.

### 2.5. Confocal Laser Scanning Microscopy (CLSM)

The microstructure of the mixed biopolymer systems was characterised using a CLSM microscope (LSM 880 with Airy scan, Zeiss, Jena, Germany) equipped with a ×10 objective. The detailed sample preparation and analysis method is described by Hellebois and co-workers [21].

### 2.6. Particle Size Distribution Measurements

The volume-weighted particle size distributions and the volume-weighted mean particle diameters (d_4,3_) of aliquots obtained from the initial food matrix and the oral, gastric, and intestinal phases obtained were determined using static laser light scattering (Mastersizer 3000, Malvern Panalytical, Malvern, United Kingdom). The refractive indexes of whey protein, sodium caseinate, and water were set at 1.45, 1.34, and 1.33, respectively.

### 2.7. Protein Digestibility

The cleavage of the intact milk proteins into peptides during the in vitro gastrointestinal processing steps was monitored by SDS-PAGE analysis adopting the protocol described by Hellebois and co-workers [23]. Prior to the analysis, all systems were diluted to 5, 5, 10, and 20 µg of proteinaceous matter per well for the initial food matrix, and oral, gastric, and intestinal chymes, respectively. The samples, XT sample buffer (Bio-Rad, Hercules, CA, United States), and 0.6 XT reducing agent (Bio-Rad) were mixed at a 1:0.25:0.05 ratio, heat treated at 95 °C for 5 min, and then transferred into the SDS-PAGE gel wells.

### 2.8. Protein Hydrolysis Quantification

The amino acid release throughout the in vitro digestibility was assessed by the o-phthaldialdehyde (OPA) method carefully described by Hellebois and coworkers [21]. The degree of hydrolysis of the protein, after considering each dilution step, was determined as follows:

(1) $$\mathrm{DH}\;(\%)=\frac{{\mathrm{NH}}_{2}{}_{\mathrm{digested}}-{\;\mathrm{NH}}_{2}{}_{\mathrm{FM}}}{{\mathrm{NH}}_{2}{}_{\mathrm{total}}-{\;\mathrm{NH}}_{2}{}_{\mathrm{FM}}}\times 100$$

where DH is the degree of hydrolysis, and ${\mathrm{NH}}_{2}{}_{\mathrm{digested}}$, ${\mathrm{NH}}_{2}{}_{\mathrm{FM}}$, and ${\mathrm{NH}}_{2}{}_{\mathrm{total}}$ denote the primary amino group content of the digested samples, food matrix, and hydrolysed FM aliquots, respectively.

### 2.9. Statistical Analyses

The normal distribution of the experimental data was confirmed by implementing the Shapiro–Wilk test and Q–Q plot representation normality tests. One-way ANOVA coupled with Tukey’s post-hoc means comparison test was performed to assess the significance of the tested parameters (milk protein type, FG type and content) on the in vitro digestibility data. All analysed were carried out using Origin 2019b software (OriginLab Inc, Northampton, MA, United States).

## 3. Results and Discussion

### 3.1. Rheological Behaviour and Colloidal Changes during the In Vitro Simulated Oral Processing

**Figure 1:**
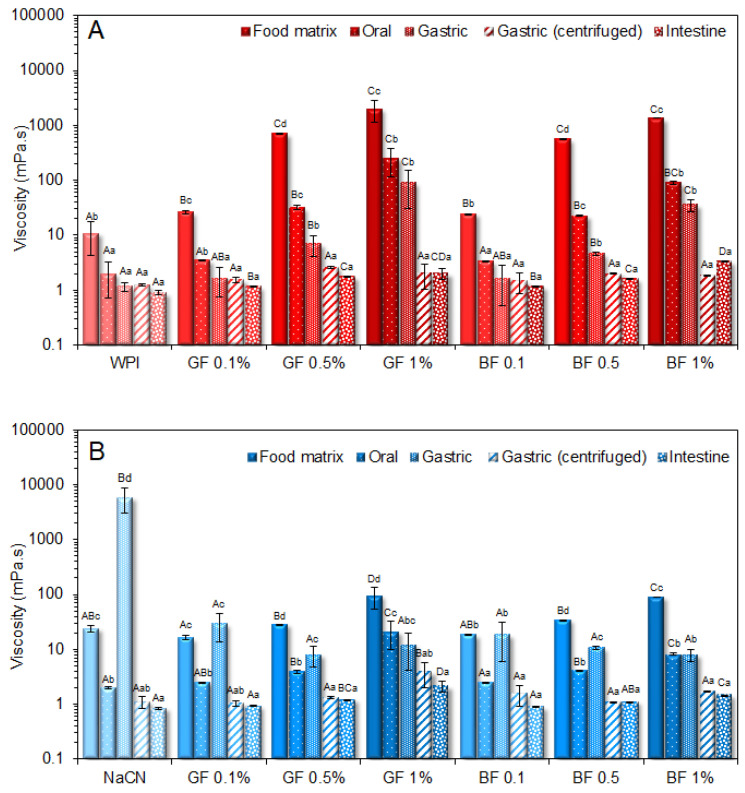
Impact of the golden (GF) and brown (BF) flaxseed gums (FG) on the apparent viscosities of the whey protein isolate (WPI) (**A**) and sodium caseinate (NaCN) (**B**) based food models and the oral, gastric, and intestine phases obtained. The term “gastric centrifuged” refers to the samples obtained following centrifugation of the gastric chymes at 4500 g for 5 min. ^a–d, A–D^ Different letters between the bars denote a significant difference among the samples differing in FG content and phenotype (uppercase) or in vitro digestion step (lowercase).

In keeping with our previous findings [21], the milk protein food models exhibited a Newtonian flow behaviour with the apparent viscosities of 23.8 and 10.8 mPa s for NaCN and WPI, respectively. Upon FG addition, a proportional increase to the FG concentration increase was observed in the apparent viscosities of the food matrices (Figure 1). No significant differences were found in the average macroviscosity values of the NaCN-based food models as a function of the FG phenotype. On average, the GF-stabilised WPI-based food models exhibited significantly (*p* < 0.05) higher viscosities than their BF counterparts (i.e., 914 and 644 mPa s, respectively). The rheological behaviour of colloidal dispersions in the presence of polysaccharides is known to be influenced by the molecular properties of the latter, such as the molecular weight, intrinsic viscosity, hydrodynamic radius, and surface charge density [24,25]. According to our previous observations [3], GF gum was characterised by a significantly higher molar mass and intrinsic viscosity (Mw = 1340 kDa, [η] = 6.6 dL g^−1^) than BF gum (Mw = 1147 kDa, [η] = 5.1 dL g^−1^). Except for the direct contribution of the polysaccharidic fraction to the viscosity of the bulk aqueous phase, the protein–polysaccharide molecular interactions e.g., electrostatic forces (electrostatic complexation or repulsion) and hydrogen bonding can also result in antagonistic or synergistic effects on the bulk rheology [26]. Although flaxseed gum is recognised as an anionic biopolymer, differences in the absolute surface charge density have been reported, i.e., |ζ| = 25–30 and 32–34 mV (pH = 7) for GF and BF gum, respectively [3]. Close to pH neutrality conditions (i.e., food models and oral boluses) implies that the observed reduction in the macroviscosity of the BF-stabilised milk protein solutions is due to the stronger repulsive forces between the BF and milk protein polymer chains. It should be noted that the macroviscosity dependence on the FG concentration followed a linear trend, with the highest slopes detected in the WPI-based solutions. Contrary to non-ionic polysaccharides, e.g., alfalfa galactomannan [21], where the macroviscosity of the milk protein solutions was radically changed at c > c*, in the FG-stabilised protein food models, it was not possible to experimentally justify such a dependence on the critical concentration (c*) i.e., c*_GF_ = 0.53 and c*_BF_ = 0.76% [3].

While the solute dilution degree plays a central role in the viscosimetric response of the oral boluses, other parameters, such as the changes in counterion composition and ionic strength upon mixing with the artificial saliva, may be equally influential. As illustrated in Figure 1, admixing the initial protein food models with the artificial saliva was associated with an approximately one-order decrease in the macroviscosity values. The milk protein type and FG phenotype had a significant impact (*p* < 0.05) on the macroviscosity of the oral food boluses obtained. Hence, the WPI systems stabilised by BF experienced the highest decrease in macroviscosity upon oral bolus formation. On the other hand, the impact of the FG concentration on the macroviscosity of the model food boluses was unclear (*p* > 0.05).

**Figure 2:**
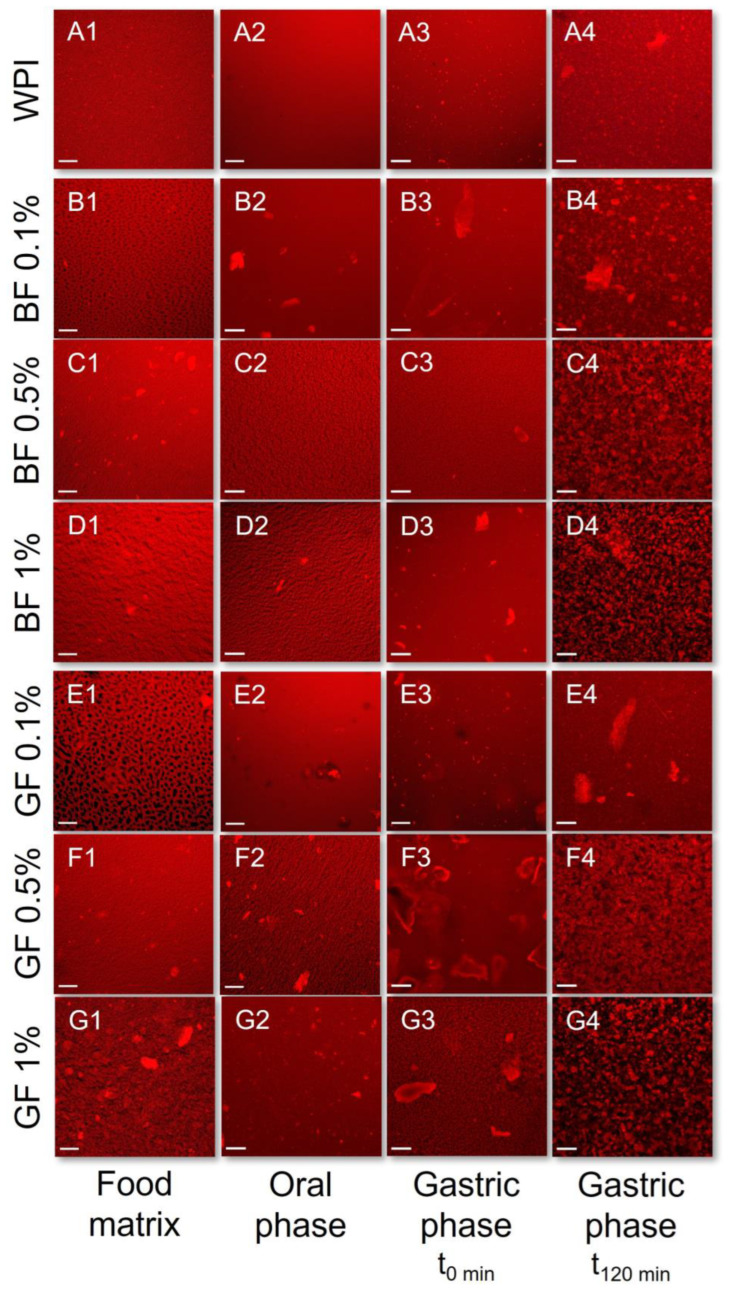
Impact of flaxseed gums on the in vitro oro-gastric induced colloidal changes of the WPI-based food models adopting the INFOGEST 2.0 in vitro digestion model. **1**: Food matrix, **2**: Oral phase; **3**: gastric (start); **4**: gastric (end); (**A**): WPI only; (**B**–**D**): WPI + BF; (**E**–**G**): WPI + GF. Scale bar = 100 μm.

**Figure 3:**
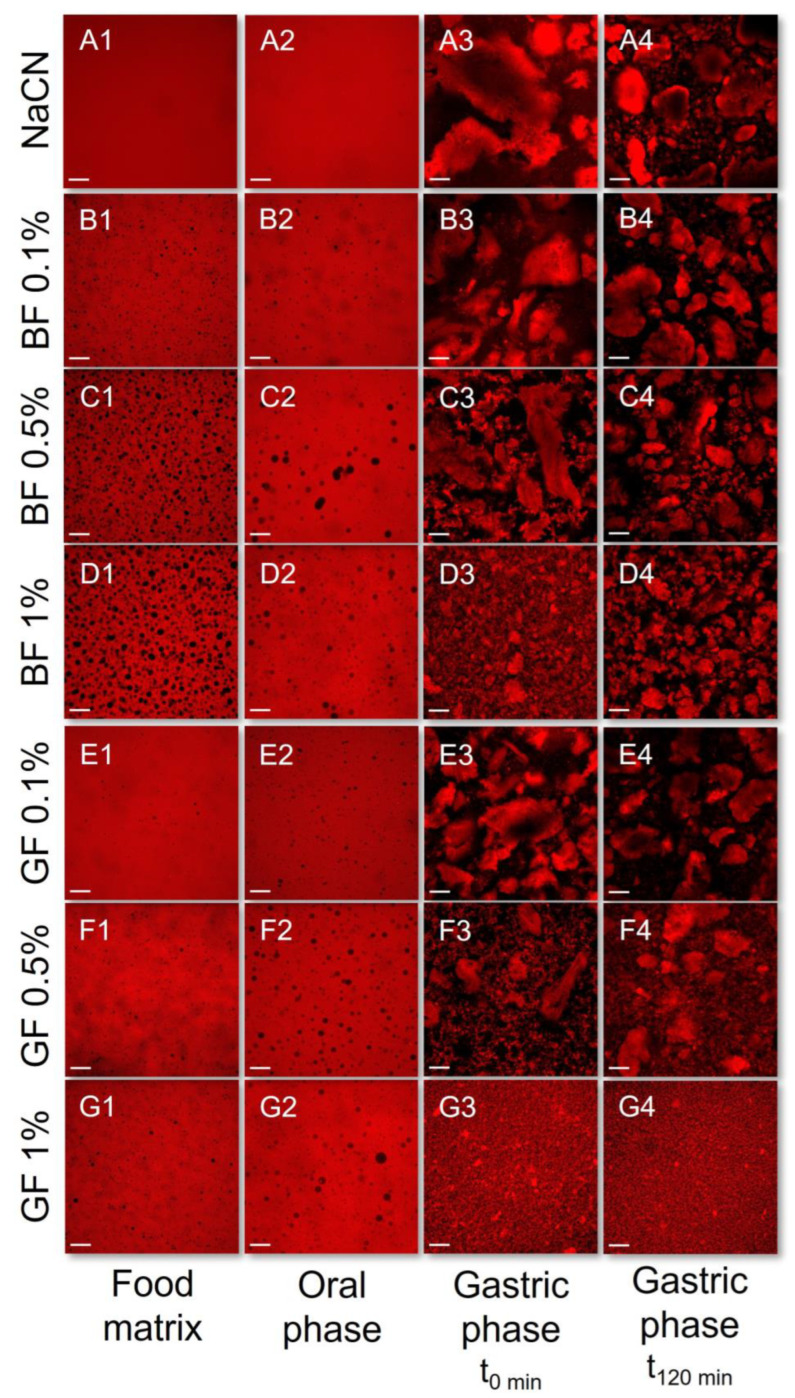
CLSM assessment of the impact of flaxseed gums on the in vitro oro-gastric induced colloidal changes of the NaCN-based food models adopting the INFOGEST 2.0 in vitro digestion model. **1**: Food matrix, **2**: Oral phase; **3**: gastric (start); **4**: gastric (end); (**A**): NaCN only; (**B**–**D**): NaCN + BF; (**E**–**G**): NaCN + GF. Scale bar = 100 μm.

**Figure 4:**
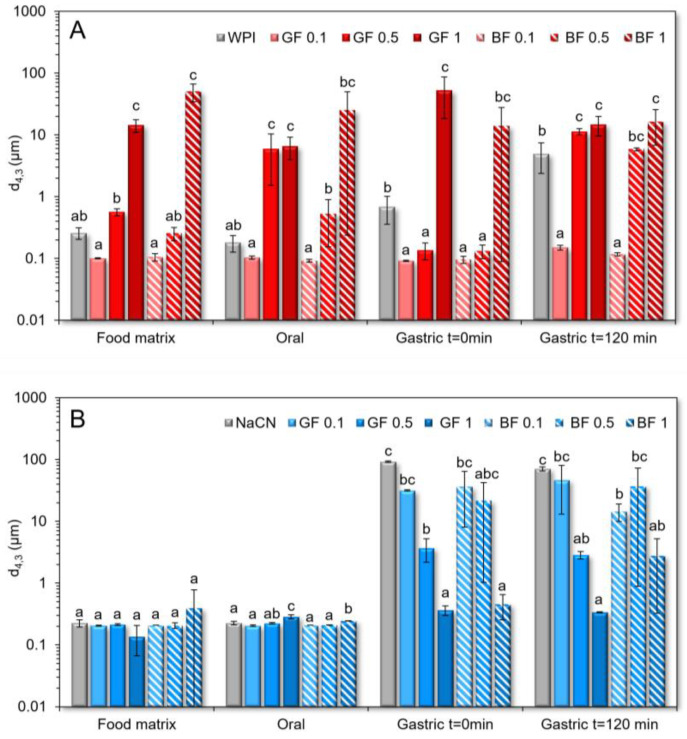
Changes in the protein aggregate particle size as they occurred throughout the simulated in vitro oro-gastrointestinal processing of the WPI-(**A**) and NaCN-(**B**) based food models. ^a–c^ Different letters between the bar phases denote a significant difference among the samples differing in the amount of gum for the same digestive.

To gain insight into the colloidal changes occurring during the oral processing, as influenced by the FG type and concentrations, the initial food models and the oral food boluses obtained were analysed by means of CLSM (Figure 2 and Figure 3) and static laser light scattering (SLS) (Figure 4). FG in the protein food models was associated with segregative microphase separation phenomena as a cause of the intermolecular repulsive forces between the FG and milk proteins [27]. From a mechanistic point of view, segregative phase separation in polysaccharide–protein aqueous systems is associated with either the Flory–Huggins or depletion flocculation theories [28]. In colloidal dispersions (i.e., biopolymer microspheres dispersed into the semi-dilute polysaccharide-rich aqueous phase), segregative phase separation is driven primarily by the depletion-flocculation mechanism, where the milk proteins behave as colloidal microspheres. Indeed, it has previously been demonstrated that both WPI and NaCN may exhibit a microsphere-like behaviour stemming from either their nano-aggregated (WPI) or rod-like self-assembled (NaCN) structure conformation [13,29].

As illustrated in Figure 2, in the WPI-based food models, a similar segregative phase separation pattern was observed between BF and GF. At c_FG_ = 0.1% wt., the WPI food models exhibited a biphasic w/w emulsion-like conformation where FG-rich water microdroplets were uniformly distributed into the continuous protein-rich aqueous phase. At c_FG_ ≥ 0.5% wt., the FG-stabilised WPI food models had a heterogenous microstructure as evidenced by the presence of protein microaggregates dispersed into the continuous phase. Given that the aggregative phenomena took place at c~c* (c*_GF_ = 0.53 and c*_BF_ = 0.76% wt. [3]), it is assumed that the transition from the dilute to the semi-dilute regime promotes the noncovalent self-association of the whey proteins [27]. To a lesser extent, electrostatic complexation between FG and WPI proteins may also occur. By adding the artificial saliva, a decisive reduction in the prominence of the demixing phenomena in the systems containing 0.1% wt. was observed (Figure 2 (B1,B2,E1,E2)), which can be ascribed to the shifting of the thermodynamic equilibrium close to the binodal curve [27]. At c_FG_ ≥ 0.5% wt., the dilution of the initial food matrices with the oral fluids resulted in a sparsely heterogeneous microstructure (Figure 2 (C1,C2,F1,F2)).

In contrast to the WPI exemplars, the NaCN-based food models exerted a demixed water-in-water emulsion-like microstructure for the whole range of c_FG_, with the BF-stabilised systems being more pronounced (Figure 3(A1–G1)). Interestingly, the emulsion-like segregated microstructure was detected in the entity of the NaCN-based oral boluses, but to a lesser extent (Figure 3(A2–G2)). No clear interrelationship between the FG concentration and the magnitude of the segregative phenomena was noted. According to the SLS measurements (Figure 4), the presence of soluble protein aggregates (d_4,3_ = 258 and 225 nm for WPI and NaCN, respectively) was confirmed. The impact of FG on the particle size of the protein aggregates was dependent on the protein type. As for the WPI food models, a proportional increase to the FG concentration increase was observed in the mean size of the soluble protein aggregates. Although the coacervation between FG and WPI at pH > pH_c,_ i.e., ~5.2–5.4 [30] is considered unlikely to occur as both biopolymers were negatively charged, the formation of soluble protein aggregates under weakly acidic conditions (pH > 5.5) has been reported, as a result of the intermolecular association between FG and WPI through oppositely charged patch junction zones [31].

Nonetheless, the FG phenotype was not influential on the mean size of the soluble protein aggregates. The addition of FG into the NaCN food models did not modify their colloidal properties for the entire range of gum concentration. In mild acidic conditions, NaCN is characterised by a significantly higher negative surface charge density than WPI [21], and hence it is assumed that the strong electrostatic repulsion between the FG and NaCN molecules hampered the formation of soluble aggregates. On admixing with the artificial saliva, none of the protein food models tested experienced any significant change in terms of the protein aggregate mean size, which implies that the changes in the ionic strength and counterion composition of the food boluses did not modify the mechanism of the FG–milk protein interactions.

### 3.2. Rheological Behaviour and Colloidal Changes under In Vitro Gastro-Intestinal Conditions

To offset the technical limitations of the static in vitro digestion models associated with the dynamics of the physiology of digestion [32], the gastric fluids were added at a constant rate and under constant stirring to achieve the target gastric pH_gastric_ = 2.5 within 5 min (t = 0 min). The enzymatic activity of pepsin was predetermined, and the amount of the HCl required for achieving the pH_gastric_ = 2.5 was customised per food model to minimise the buffering effect of the milk proteins and FG. In addition, the peptic cleavage process was hindered using a protease inhibitor cocktail, whilst a real-time analysis of the samples, stored in an ice bath, was conducted on the same day.

All gastric phases exhibited a suspension-like behaviour owing to the formation of acid-mediated protein aggregates. The viscosimetric characterisation of the gastric phases (before and after centrifugation) revealed that the NaCN-based food models experienced the highest increase in apparent viscosity at 50 s^−1^ compared to the WPI exemplars (Figure 1). Interestingly, centrifugation resulted in an abrupt (almost four-order magnitude) decline in the macroviscosity values of the NaCN-based gastric chymes, which can be attributed to the depletion of their sedimentable protein aggregate matter (Figure 1B). On the other hand, no significant differences in the macroviscosity of the actual and centrifuged WPI gastric phases were observed, indicating the presence of finer and more uniform protein aggregates (Figure 1A).

**Figure 5:**
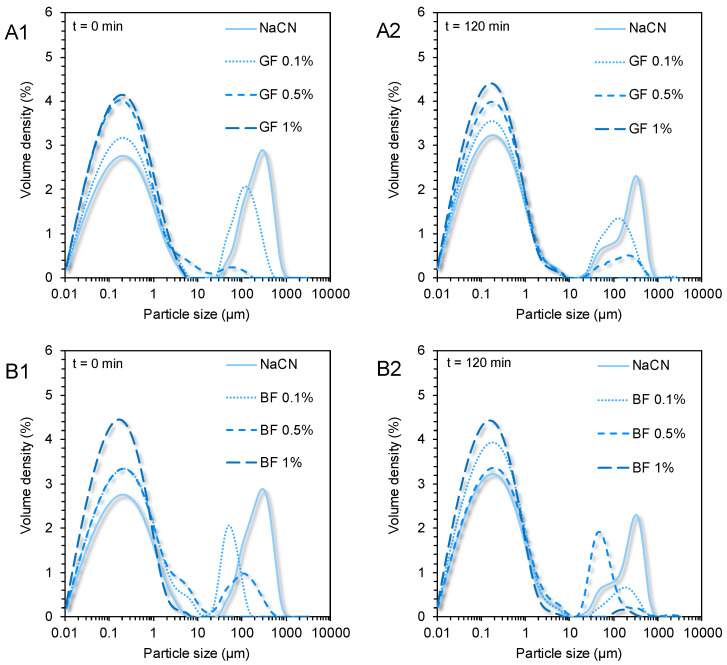
Particle size distribution profiles of the initial and end NaCN-based gastric phases (**1**: t = 0 min and **2**: t = 120 min) as influenced by the phenotype and concentration of flaxseed gum. (**A1**,**A2**) = golden flaxseed and (**B1**,**B2**) = brown flaxseed.

As well illustrated in the acquired CLSM micrographs (Figure 3) and SLS analysis data (Figure 4), the NaCN-based gastric phases comprised large (d_4,3_ = 91 μm) acid-induced protein aggregates. Although caseins may also undergo pepsin-induced clotting, in the case of NaCN, this is considered unlikely owing to its nonmicellar structure conformation [6]. The bimodal pattern of the particle size distribution curves of the NaCN-based gastric chymes is generally confirmatory of the data in the literature [15,20,21]. At the end of the gastric processing step, a reduction in the d_4,3_ values (i.e., d_4,3_ = from 91 to 71 μm) throughout digestion was observed as a result of the peptic cleavage and the in vitro simulating mechanical (antral) forces. The presence of increasing FG content was associated with a proportional reduction in the mean size of the NaCN aggregates, as demonstrated by the decay in the intensity of the above-micron particle population peak (Figure 5). Nonetheless, no significant differences in the d_4,3_ values were observed at the start and endpoint of the in vitro gastric processing (i.e., 15.7 and 17.3 μm, respectively). Interestingly, the decay in the d_4,3_ values followed an exponential trend as follows:

d = d_0_ + A⋅exp (−k_c_⋅c_FG_) (2)

where d, d_0_ denote the d_4,3_ values (in μm) of the NaCN aggregates in the absence (d_0_) and presence of FG (d), A is a constant, and k_c_ is the NaCN aggregate size reduction rate (%^−1^) as a function of the FG content. 

According to our calculations, the k_c_ values at t = 0 min were 11.02 ± 0.82 and 11.42 *±* 0.99%^−1^ for GF and BF, respectively, which were significantly higher than the corresponding ones at t = 120 min (i.e., 4.54 ± 0.51 and 4.18 ± 0.73%^−1^ for GF and BF, respectively). This suggests that the FG impeded the acid-induced aggregation responsiveness of NaCN to the gastric fluids resulting in smaller and more uniform particle size distributions. Indeed, this was confirmed by employing the CLSM (Figure 3) and SLS measurements (Figure 5). Although the mechanism was not evidenced, it can be assumed that the colloidal changes observed were governed by the ability of FG to adsorb onto the NaCN particles via electrostatic complexation upon acidification, and its direct effect on the continuous aqueous phase macroviscosity of the gastric chymes, which diminished the diffusion rate of pepsin into the solid (protein aggregates)–water interface [15,20,21].

**Figure 6:**
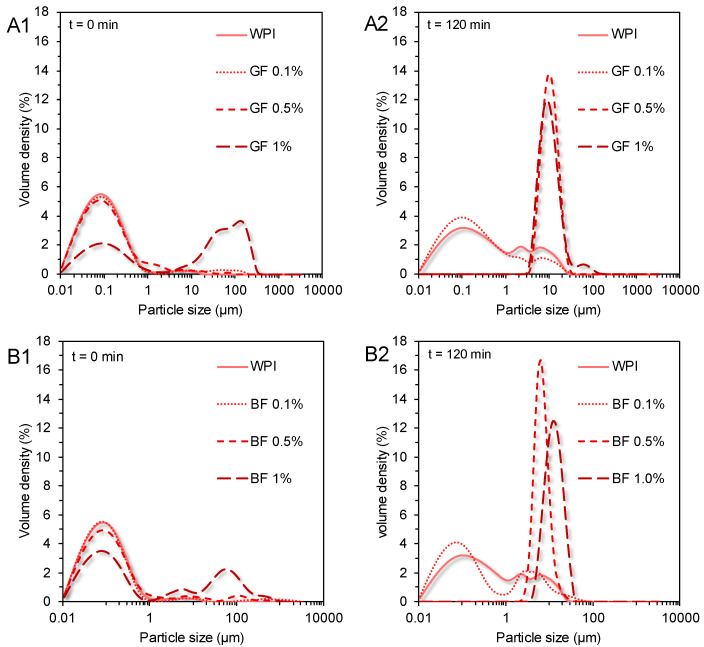
Particle size distribution profiles of the initial and end WPI-based gastric phases (**1**: t = 0 min and **2**: t = 120 min) as influenced by the phenotype and concentration of flaxseed gum. (**A1**,**A2**) = Golden flaxseed and (**B1**,**B2**) = Brown flaxseed.

In opposition to the beforementioned findings, the initial WPI-based gastric phases exhibited a relatively uniform microstructure (d_4,3_ = 0.69 µm), in agreement with our previous observations [21]. In the absence of FG, a bimodal particle size distribution pattern was confirmed in the WPI exemplars. A significant increase in WPI acid-formed aggregates (d_4,3_ = 4.9 μm) was detected at the end of the gastric processing step. Markussen and co-workers [20] have reported a similar behaviour in semi-dynamically digested milk protein concentrate (MPC)-based food models. Upon mixing with the gastric fluids, all gastric phases containing FG were characterised by highly polydisperse colloidal suspensions (Figure 6). Despite the positive correlation between the d_4,3_ values and the c_FG_, the mean size of the particles was substantially changed only at c_FG_ = 1% wt. (Figure 4). GF addition resulted in the formation of the largest protein aggregates, which may be associated with its higher negative surface charge density in highly acidic conditions (i.e., pH = 2–4) than BF [3], favouring its electrostatic bridging with whey proteins. Throughout gastric processing, a re-organisation of the morphological features of the WPI-FG gastric phases was observed, as illustrated in the CLSM micrographs (Figure 2). As seen in Figure 6, the submicron fraction of the particles (representing the WPI nanoaggregates colloidal dispersion), was decreased at the expense of the fraction larger than a micron, i.e., 1–50 μm, when c_FG_ > 0.5% wt. This implies that the formation of WPI–FG coacervates took place throughout gastric processing leading to the progressive depletion of the colloidal WPI soluble aggregates and, eventually, to the increase of the sedimentable biopolymer matter. Indeed, the centrifugation of the gastric endpoint phases resulted in a significant decrease in their apparent viscosities (Figure 1). Omitting the discrepancies due to ionic strength, the macroviscosity of the centrifuged gastric phases (i.e., 1.6–2.6 mPa s) was significantly lower than that of the dilute flaxseed gum solutions (i.e., 9.5–17.6 mPa s [3]). Hence, the colloidal changes in the WPI-FG gastric phases are driven primarily by an electrostatic complexation mechanism.

### 3.3. In Vitro Digestibility of the Milk Proteins

**Figure 7:**
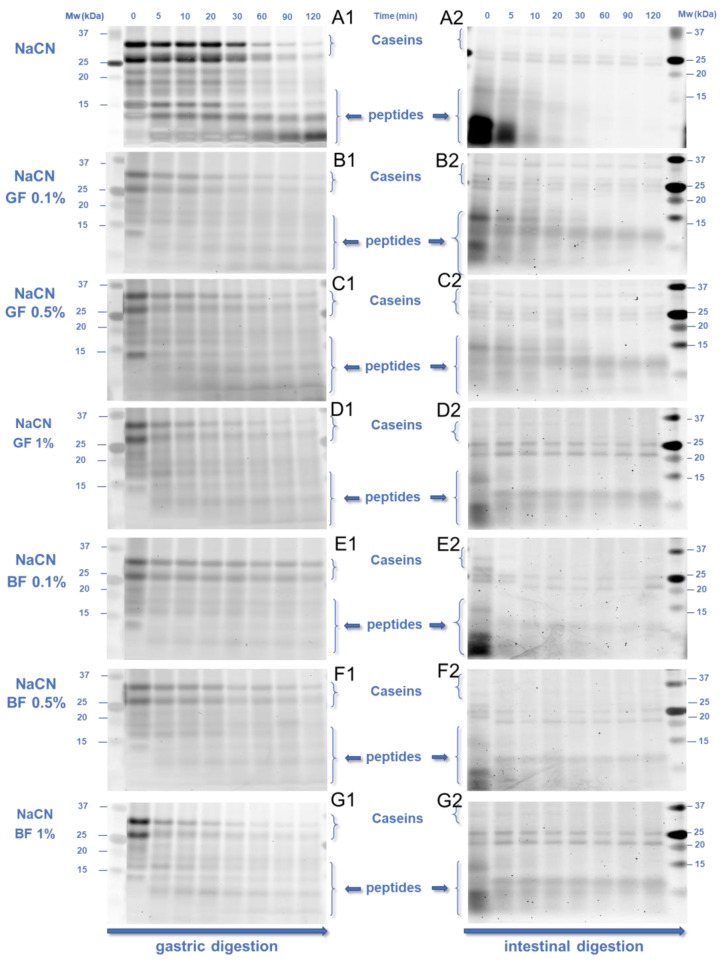

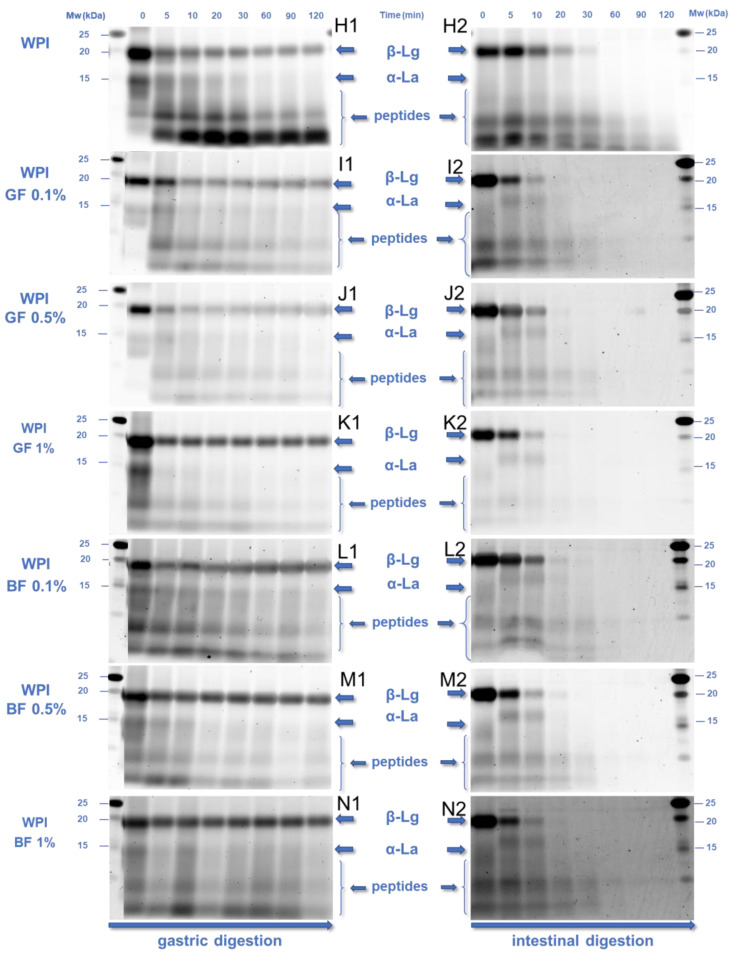
SDS-PAGE electropherogram of the gastric (**1**) and intestinal (**2**) phases of the NaCN-(**A**–**G**) and WPI-(**H**–**N**) based food models as influenced by the phenotype and concentration (0.1–1% wt.) of flaxseed gum. An SDS-PAGE analysis was conducted on gastric and intestine aliquots sampled at predetermined time points (0, 5, 10, 20, 30, 60, 90, and 120 min). Abbreviations used: β-Lg: β-lactoglobulin, α-La: α-lactalbumin.

**Figure 8:**
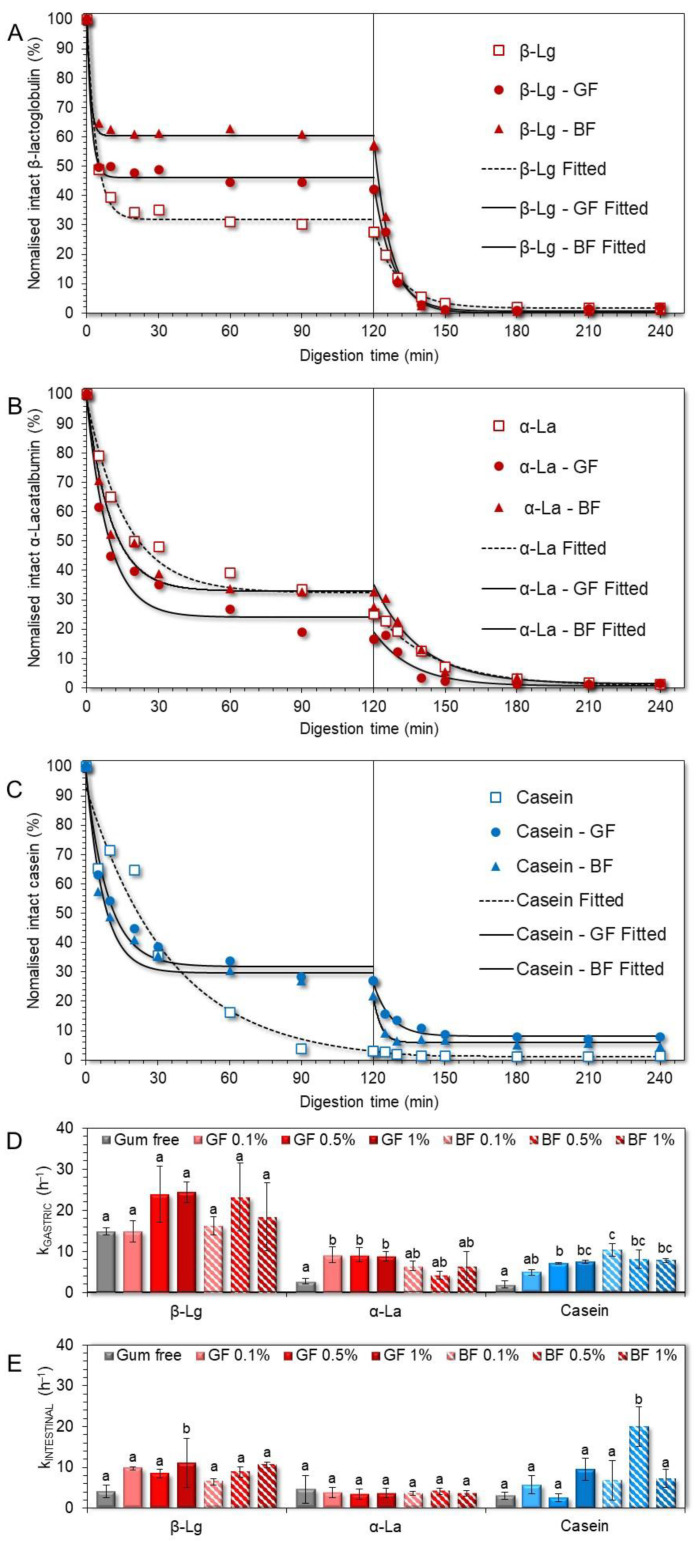
SDS-PAGE densitometric analysis illustrating the kinetics of β-lactoglobulin (**A**), α-lactalbumin (**B**), and total caseins (**C**) fate in the gastric (t = 0−120 min) and intestinal (t = 120−240 min) phases as influenced by the phenotype and concentration of flaxseed gum. The calculated rates for β-Lg, α-La and casein are depicted in (**D**) (gastric rates) and (**E**) (intestinal rates). ^a–c^ Different letters for the same protein type denote a significant difference among the samples. Abbreviations used: β-Lg: β-lactoglobulin, α-La: α-lactalbumin.

**Figure 9:**
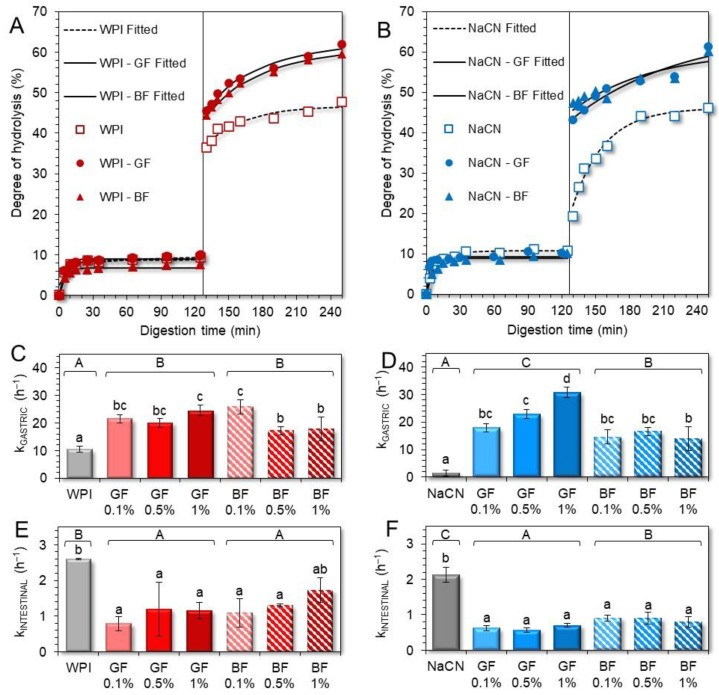
Degree of protein hydrolysis in the WPI-(**A**) and NaCN-(**B**) based gastric and intestinal phases as influenced by the phenotype and concentration (0.1–1% wt.) of flaxseed gum. The protein hydrolysis rates of WPI and NaCN during gastric (k_GASTRIC_) and intestinal (k_INTESTINAL_) steps are displayed in (**C**–**F**). ^a–d, A–C^ Different letters between the samples denote a significant difference.

The peptic cleavage of milk proteins and the degree of proteolysis were measured employing SDS-PAGE (Figure 7 and Figure 8) and OPA (Figure 9) assay, respectively. To gain insight into the kinetics of the peptic cleavage of the milk proteins, the SDS-PAGE images obtained (Figure 7) were analysed densitometrically using ImageJ software (Figure 8), as previously reported by Hellebois and co-workers [21]. Different aliquots of the oral, gastric, and intestinal phases (i.e., 5, 10, and 20 μg of proteinaceous matter per well) were used to improve the detection of the intact and fragmented proteins. Three significant bands identified at 25–35, 18.2, and 14.2 kDa allotted to total caseins (a_s_−, β−, and κ−casein), β-lactoglobulin (β-Lg), and α-lactalbumin (α-La) were semi-quantified and the densitometric data were fitted to an exponential decay model [21,33,34], as follows:

(3) $${c=c}_{120}+\left(c_{0}-{\;c}_{120}\right)\exp(-\mathrm{kt})$$

where c_0_ and c_120_ denote the normalised percentage of the residual intact protein at the beginning or end of the gastric or intestinal processing step, t is the gastric or intestinal processing time (in h), and k (h^−1^) is the peptic cleavage rate of proteins to polypeptides. The time τ (in h) required to cleave the 50% of the initial proteins was calculated according to the half-time (for first-order kinetics) equation:

(4) $$\tau =\frac{\ln2}{k}$$

In the absence of FG (Figure 8D), β-lactoglobulin underwent a faster peptic cleavage during the gastric processing step than α-lactalbumin and total caseins (k = 14.9 ± 0.9, 2.7 ± 0.7 and 1.9 ± 0.8 min^−1^, respectively). Besides the structure conformational aspects of the proteins [35], several processing parameters associated with the implementation and severity of heat treatment, pressure processing, enzymatic pre-treatment, etc., may affect the sensitivity of milk proteins to peptic cleavage [9,10,36,37]. According to Figure 8A–C, the presence of FG was associated with a higher amount of intact β-lactoglobulin (42−57%) and total caseins (22−27%) and a lower amount of intact α-lactalbumin (16−28%), at the end of the gastric processing step.

It is presumed that the differences in the pepsin-induced cleavage of whey proteins may stem from the differences in their secondary structure conformation and surface charge density. β-Lactoglobulin, due to its higher isoelectric point (pI_β-Lg_~5.2, pI_α-La_~4.6), can undergo electrostatic complexation with FG earlier than α-lactalbumin, leading to the steric hampering of the peptic cleavage in the former case. From a kinetic perspective, α-lactalbumin and total caseins underwent pepsin-induced peptic cleavage at a slower pace (τ = 15.2 and 21.4 min, respectively) than β-lactoglobulin (τ = 2.8 min, Figure 8D). In the presence of FG, the time required for cleaving the 50% of the initial intact proteins was shortened (τ = 2.1, 6.1, and 5.7 min for β-lactoglobulin, α-lactalbumin, and total caseins, respectively), although its phenotype did not play any significant role (Figure 8D). The ability of the polysaccharides to modify the kinetics of the pepsin cleavage has been reported in previous studies [15,16,17,20,21]. For anionic polysaccharides, the major mechanistic pathway associated with the reduced digestibility of milk proteins concerns their direct molecular interactions that sterically impede the access of proteases to the cleaving sites of the protein substrate [15,20]. In addition, some anionic polysaccharides i.e., sodium alginate, can selectively inhibit the enzymatic activity of proteases (i.e., pepsin) [38]. Although these results could lead us to speculate to some extent on the pepsin-inhibiting capacity of FG, further experiments are required in order to confirm this hypothesis.

By monitoring the temporal changes in the intact protein residue in the intestinal phases, an almost complete cleavage of the milk proteins was detected as less than 2–3%, and 10% of intact whey proteins and total caseins remained at the end of the intestinal processing. Based on the calculated kinetic parameters (Figure 8E), no significant differences in the time required for cleaving the 50% of the intragastric residual intact protein matter were found i.e., τ = 10.3, 9.3, and 13.7 min for β-lactoglobulin, α-lactalbumin and total caseins, respectively. The presence of FG did not modify significantly the peptic cleaving rate of α-lactalbumin (i.e., τ = 11.3 min). On the other hand, the pancreas mediated cleaving rates of β-lactoglobulin and total caseins were increased (i.e., τ = 4.7 and 7 min, respectively); however the differences observed were nonsignificant.

In order to better understand the impact of the FG phenotype and content on the extent of proteolysis throughout the in vitro simulated gastric and intestinal processing steps, the data obtained from the OPA assay were fitted to the following mathematical model, as previously reported by Le Feunteun and co-workers [33]:

(5) $$\mathrm{Gastric}\;\mathrm{phases}:{\mathrm{DH}}_{\mathrm{gastric}}{=\mathrm{DH}}_{\mathrm{gastric},\;\infty }\exp(1-k_{\mathrm{gastric}}t)$$

where the DH_gastric,∞_ denotes the degree of hydrolysis (%) at the end of the gastric processing and k_gastric_ is the rate of hydrolysis (in h^−1^), and t the digestion time.

(6) $$\mathrm{Intestinal}\;\mathrm{phases}:{\mathrm{DH}}_{\mathrm{intestinal}}{=\mathrm{DH}}_{\mathrm{intestinal},120}+\left({\mathrm{DH}}_{\mathrm{intestinal},0}-{\mathrm{DH}}_{\mathrm{intestinal},\;120}\right)\exp(-k_{\mathrm{intestinal}}t)$$

where the DH_0,_ and DH_120_ denote the degree of hydrolysis (%) achieved at the beginning and end of the intestine processing step, k_intestinal_ is the rate of hydrolysis (in h^−1^), and t the digestion time.

As displayed in Figure 9A,B, the extent of hydrolysis (% DH) achieved at the end of the gastric processing step was at most 9–11%, regardless of the milk protein type and FG presence. It should be noted that in all gastric chymes, the calculated hydrolysis rates were highly dependent on the changes in the amount of the free amino groups during the very early stages of the gastric processing i.e., within the first 30 min. Similar behaviour in the digestion of food biomacromolecules as influenced by the food matrix characteristics has been reported [39]. In line with previous studies [21,36,40], the rate of hydrolysis in the WPI only gastric phases was substantially higher than their NaCN only counterparts (Figure 9C,D). This is generally associated with the restricted colloidal responsiveness of WPI to pH and ionic strength changes that sterically favour the peptic cleaving activity of pepsin.

Incorporating FG into the initial food models induced a significant increase in the hydrolysis rates achieved during the gastric processing step, i.e., k_gastric_ = 10.5 ± 1.1 and 21.1 ± 3.5 h^−1^ (for WPI-based chymes in the absence and presence of FG, respectively) and 1.5 ± 1.1 and 19.4 ± 6.4 h^−1^ for their NaCN counterparts. This can be ascribed to the ability of FG to control the acid-induced aggregation of the milk proteins and, therefore, the accessibility of pepsin to the protein substrate. The pepsinolysis-modulating role of FG was mainly flaxseed phenotype-driven (Figure 9C,D). In this context, the GF-stabilised food models underwent faster pepsinolysis (k_gastric_ = 22 ± 1.9 and 23.9 ± 5.7 h^−1^ for the WPI and NaCN-based food models, respectively) than their BF exemplars (k_gastric_ = 20.3 ± 3.9 and 15 ± 1.0 h^−1^ for the WPI- and NaCN-based food models, respectively). However, only in the case of NaCN the differences in the rates of hydrolysis were significant (*p* < 0.05). Noteworthily, the k_gastric_ rates obtained hereby were significantly higher than those observed in the case of non-ionic polysaccharides (i.e., alfalfa galactomannan) [21]. Hence, it appears that the ability of FG to restrict the ripening of the acid-mediated protein aggregates via the formation of electrostatic complexes of controlled mean size is significantly more influential than its direct contribution to the solid–liquid interface mass transfer phenomena. In keeping with the latter, the modulating role of FG concentration in the induced pepsinolysis was unclear as it was only in the case of the GF-stabilised NaCN food models that there was a proportional increase to the gum content increase in hydrolysis rates.

On the completion of the intestinal digestion, a substantial increase in the DH of the proteins was observed i.e., ca. 47% for individual milk proteins and 59–62% when FG was incorporated in the milk protein-based food models (Figure 9A,B). Nonetheless, the pancreas-mediated hydrolysis rates were significantly (p < 0.05) lower in the intestinal digesta containing FG (Figure 9E,F). Although the hydrolysis rates were higher in the intestinal phases containing BF (k_intestinal_ = 1.37 and 0.86 h^−1^ for the WPI- and NaCN-based food models, respectively) than their GF counterparts (k_intestinal_ = 1.05 and 0.63 h^−1^ for the WPI- and NaCN-based food models, respectively), the differences were nonsignificant. Our findings postulate that the equilibrium between the coacervates and precipitates, free proteins, and polyanions had a substantial impact on the extent of pancreas-induced hydrolysis [41,42]. Upon admixing of the gastric chymes with the simulating intestinal fluids, the concomitant increase in the pH (from 2.5 to 7.0), ionic strength, and cationic species (Na^+^, K^+^, and Mg^2+^) resulted in the progressive dissolution of the milk protein-FG electrostatic complexes and the release of free proteins that can be cleaved more easily from the pancreas. At this stage, the solubilisation kinetics of the coacervates, together with the impact of the thickening capacity of the FG on the interphase mass transfer kinetics, became more influential on the rate of the polypeptide peptic cleavage.

## 4. Conclusions

In the present work, the ability of FG to modulate the colloidal transformation of milk proteins and, consequently, their cleavage throughout in vitro digestion was elucidated. Regardless of its phenotype, FG was associated with segregative phase separation in both tested milk protein-based food models. Upon oral processing, only the WPI-based food boluses were colloidally responsive to FG due to the formation of soluble protein-gum complexes. Under intragastric simulating conditions, both milk proteins underwent acid-induced aggregation with the colloidal changes (mean particle size and morphological aspects) in the NaCN-based systems being critically influenced by the presence of FG; however, the FG phenotype played a minor role. On the completion of the gastric processing, the presence of FG was associated with a higher amount of residual intact total caseins and β-lactoglobulin but it did not significantly impact the intact α-lactalbumin residue. No remarkable differences in the amount of the residual intact proteins at the end of the intestinal digestion were observed. Although the FG did not remarkably influence the degree of hydrolysis during the gastric processing step, FG resulted in a significant increase in the degree of hydrolysis in the intestinal digesta obtained. From a mechanistic point of view, the equilibrium between the FG-milk protein coacervates and free polyelectrolytes, as influenced by the physicochemical changes occurring during the gastrointestinal transit, is the principal modulator of the in vitro digestibility of the milk proteins. To a lesser extent, the ability of FG to increase the microviscosity in the solid–liquid interphase can also affect the sensitivity of milk proteins to peptic cleavage, particularly in pH conditions where the formation of insoluble protein–polysaccharide complexes is not favoured.

## Data Availability

The data presented in this study are available on request from the corresponding author.
